# Medical Schools of the Valley of the Mississippi

**Published:** 1838

**Authors:** 


### Art. XII.—Medical Schools of the Valley of the Mississippi.

| | | | | |
|---|---|---|---|---|
| Transylvania University, | 227 | pupils, | 64 | graduates. |
| Cincinnati College, | 125 | " | 23 | " |
| Louisville Medical Institute, | 80 | " | 24 | " |
| Medical College of Ohio, | 80 | " | 15 | " |
| Willoughby University, | 40 | " | | |
| Medical College of Louisiana, | 25 | conjectural, | | |
| | 567 | | | |

While this aggregate is within 6 or 8 of what it was for the winter of 1836-7, the addition to the Cincinnati College is 40; a satisfactory evidence of present growth, and an encouraging guarantee for the future. Our Atlantic brethren will perceive, that the West is seriously engaged in educating her own physicians. May she aim at excellence of instruction, still more than relative increase of numbers. D.

